# The Design, Development and Evaluation of the Vegetarian Lifestyle Index on Dietary Patterns among Vegetarians and Non-Vegetarians

**DOI:** 10.3390/nu10050542

**Published:** 2018-04-26

**Authors:** Lap T. Le, Joan Sabaté, Pramil N. Singh, Karen Jaceldo-Siegl

**Affiliations:** 1 School of Public Health, Loma Linda University, Loma Linda, CA 92354, USA; 2 Center for Nutrition, Healthy Lifestyle and Disease Prevention, Loma Linda University, Loma Linda, CA 92354, USA; jsabate@llu.edu (J.S.); kjaceldo@llu.edu (K.J.-S.); 3 Center for Health Research, Loma Linda University, Loma Linda, CA 92354, USA; psingh@llu.edu

**Keywords:** vegetarian lifestyle index, VLI score, dietary patterns, Adventist Health Study, AHS-2

## Abstract

Traditionally, healthful diets and lifestyles have been examined only in relation to single nutrients, foods, or food groups in terms of dietary exposure. An alternative approach is to conceptualize an index based on vegetarian food pyramid guidelines as a measure of overall diet and lifestyle quality. Our objectives were to: (1) develop the Vegetarian Lifestyle Index (VLI); and (2) evaluate adherence to the Vegetarian Food Guide Pyramid (VFGP) among a low-risk population of Adventists. The index was based on the operationalization of 14 dietary and lifestyle components. All components were equally weighted. Higher score reflected greater adherence to the VFGP. The analytic sample (*n* = 90,057) comprised 47.7% non-vegetarians, 5.6% semi-, 10.1% pesco-, and 29.0% lacto-ovo-vegetarians, and 7.7% vegans, of which 1.1% were current smokers and 9.9% were alcohol consumers. Population mean VLI score was 7.43 (SD = 1.75) ranging from 1 to 12.5. Non-vegetarians (6.14; 95% confidence interval (CI), 6.06–6.21) had a significantly lower mean compared to semi- (7.31; 95% CI, 7.22–7.40), pesco- (7.41; 95% CI, 7.32–7.49), and lacto-ovo-vegetarians (8.16; 95% CI, 8.08–8.24), as well as vegans (8.88; 95% CI, 8.78–8.96). Vegetarians scored on average 1.18 to 2.73 more points than their non-vegetarian counterparts. Results demonstrate that the index has strong discriminant ability across distinct dietary patterns. Additionally, the VLI provides a useful measure of diet and lifestyle adherence to further refine vegetarian food pyramid guidelines.

## 1. Introduction

While independent epidemiologic research continues to underscore the benefits of plant-based diets, the overarching goal is to strengthen the connection between diet, lifestyle, and health outcomes. The concept of a diet–lifestyle index as a measure of nutrition-related exposure has recently emerged in nutritional epidemiology. The purpose of the index is to compile large amounts of information into a single metric score by characterizing individuals according to the extent in which their dietary and lifestyle practice is ‘healthy’. Using an index offers a perspective that is different from the traditional approach. Prior development of these indices was directed at evaluating single nutrients [1,2,3,4,5], foods, and combination of foods based primarily on dietary recommendations [6,7,8,9,10,11,12,13,14]. Many of these indices were developed to assess compliance with national dietary guidelines, such as the Dietary Guidelines for Americans (DGA) [15,16,17,18,19,20,21], Dietary Approaches to Stop Hypertension (DASH) [17,22,23,24], and the Mediterranean Diets [19,21,25,26,27], with the goal to promote health and disease prevention. The World Cancer Research Fund/American Institute for Cancer Research (WCRF/AICR) index [28,29,30,31,32] incorporates both diet and lifestyle components based on WCRF recommendations for cancer prevention. Currently, no epidemiologic studies have attempted to conceptualize a global index based on vegetarian dietary patterns and guidelines as a systematic approach to assessing overall diet and lifestyle quality. 

Thus, the objectives of this study were: (1) to design and develop the Vegetarian Lifestyle Index (VLI) based on the Loma Linda University (LLU) Vegetarian Food Guide Pyramid [33], which consists of both diet and lifestyle recommendations; and (2) to evaluate the VLI for dietary patterns among vegetarians and non-vegetarians in the Adventist Health Study-2 (AHS-2) cohort. The AHS-2 provides an ideal opportunity to examine this connection, due to its wide range of plant food consumption and lifestyle practices. 

## 2. Materials and Methods

### 2.1. Study Population

Adventist Health Study 2 (AHS-2) is an on-going prospective study, which includes 96,469 subjects aged 30–112 years living in the USA and Canada, who completed a comprehensive questionnaire from enrollment in 2002. This population is unique in its diverse dietary and lifestyle behaviors. Adventists in the USA are mostly non-smokers. Most do not consume alcohol, or they do so infrequently [34]. There is a large variability in the consumption of plant foods, such as nuts, soya, legumes, and grains [35], and many are vegetarians (i.e., pesco-, lacto-ovo-vegetarian, and strict vegetarians). A little less than half (as compared to the general US population) are non-vegetarians [36]. We excluded subjects if data for questionnaire return dates, date of birth, sex, and race were missing; if energy intake was <500 kcal/day or >4500 kcal/day, and/or if body mass index (BMI) was <15 and >45. We also exluded questionnaires with improbable response patterns such as identical responses to all questions on a single page, or more than 69 missing values in dietary data. Guided multiple imputation was performed on the remaining observations of dietary variables with less than 10% missing values. Application of multiple imputation and exclusion criteria left an analytic sample of 90,057 participants. Written informed consent was obtained from all participants upon enrollment, and the study protocol was approved by the institutional review board of Loma Linda University.

### 2.2. Dietary and Lifestyle Data

Dietary and lifestyle behaviors were assessed using a self-administered lifestyle and health questionnaire which includes a quantitative food frequency questionnaire (FFQ), questions about physical activity, smoking status, alcohol intake, past medical history, family history of cancer, supplementations, and demographics [34]. The dietary component is the largest part of the questionnaire, and consists of questions with respect to 130 foods/types of food, frequency of consumption, and portion size. Each hard-coded item captures frequency and portion size, including 8–9 frequency categories ranging from “never consume” to “more than once each day”. Serving size is reflected in three categories ranging from standard serving, ½ or less servings, and 1½ or more cups, tablespoons, or slices depending on the food type. Additionally, write-in items are included to reflect foods consumed by participants that are not included in the food list with similar options for frequency and portion size. All dietary data were entered using the Nutrition Data System for Research (NDS-R), and food composition data were based on the NDS-R 2008 database [35]. 

Dietary patterns were characterized by reported intake of five food groups (red meat, poultry, fish, dairy, and eggs) [37]. Non-vegetarians are defined as those who consume all meats combined including fish at least once per week. Semi-vegetarians are defined as consuming red meat and poultry once per month or more, and all meats including fish once per month or more, but no more than once per week. Pesco-vegetarians consume fish once per month or more but all other meats less than once per month. Lacto-vegetarians consume eggs and dairy once per month or more, but fish and other meats less than once per month. Lastly, vegans or strict vegetarians are defined as those who consume eggs, dairy, fish, and all other meats less than once per month. A comparison of FFQ intake estimated against multiple 24-h dietary recalls for several foods is reported elsewhere [35]. For the five food groups relevant to dietary pattern characterization, correlations were moderate to high.

### 2.3. Loma Linda University Vegetarian Food Guide Pyramid

**Figure 1:**
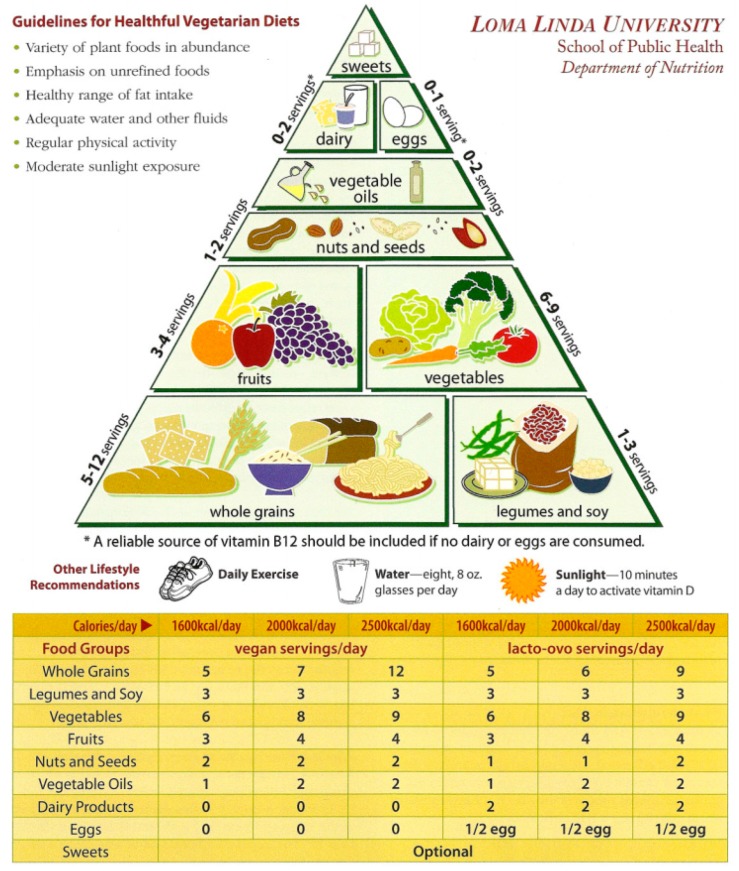
The Vegetarian Food Guide Pyramid Guidelines for Healthful Vegetarian Diets.

The various health benefits of the vegetarian diet have been established in the research literature from prospective cohort studies [38,39] to randomized clinical trials [40,41,42]. In light of the evidence, a food guide was developed for vegetarians and vegans such that a well-planned vegetarian diet could achieve nutrient adequacy, and was presented at the Second International Congress on Vegetarian Nutrition in 1994 [43]. It should be noted that these guidelines were detemined from nutrient analysis of constructed food groups. In 1999, the Vegetarian Food Guide Pyramid (VFGP) graphic image [33] was developed at Loma Linda University School of Public Health to accompany the vegetarian food guide. The VFGP was presented at the Third International Congress on Vegetarian Nutrition to provide a framework for future work to refine dietary guidelines for optimal healthful vegetarian diets to achieve nutrient adequacy and promote health. Figure 1 outlines the 13 main recommendations and one on the implicit avoidance of flesh-food intake for healthful vegetarian diets and active lifestyle. Five major plant-based food groups form the base of the trapezoidal-shaped pyramid [44]. These include whole grains, legumes and soy, fruits and vegetables, and nuts and seeds. Four optional food groups, including vegetable oils, dairy, eggs, and sweets contribute to the upper portion of the pyramid. An additional recommendation is added to address intakes of vitamin B-12 from reliable sources, including fortification or supplementation, if no dairy or eggs are consumed. Other lifestyle recommendations consist of daily exercise, water intake, and moderate exposure to sunlight. 

### 2.4. Design and Construction of Vegetarian Lifestyle Index (VLI)

The Vegetarian Lifestyle Index (VLI) consists of 14 components: 11 on diet and 3 on lifestyle behaviors. Unlike other diet-based indices, the VLI includes additional lifestyle components. Nine of the 14 components were assessed for adequacy: whole grains; legumes, soy and meat substitutes; vegetables; fruits; nuts and seeds; reliable sources of vitamin B-12; daily exercise; water intake; and sunlight exposure. Five components were assessed for moderation of intake: sweets, vegetable oils, dairy, eggs, and flesh-food intake (i.e., red meat, poultry, and fish). For the adequacy components, dietary intake or lifestyle activity at the level of the recommended amount or higher received the highest score. In contrast, for moderation components (to be consumed in limited amounts), increasing intake received lower scores. Specific cutoffs were selected based on predefined recommendations of the LLU Vegetarian Food Guide Pyramid and/or current literature. Because each component reflects an aspect of diet quality and lifestyle practice, these aspects can be added together to produce a composite score.

#### Operationalization of the VLI Scores

We used data from the AHS-2 diet and lifestyle questionnaire to operationalize the cut-off points for each of the 14 components. First, estimates of nutrients, foods, or food group intake, as well as lifestyle practice, were assessed. Food and nutrient intake were estimated using the product–sum method [45] and standardized to the 2000 kcal per day specific to the lacto-ovo-recommendations.

Secondly, predefined cut-off values were applied to develop the index scores for each component. For adequacy components, participants received a respective score of 1, 0.5, or 0 points when recommendation was met, half met, or not met. For example, the LLU Vegetarian Food Guide Pyramid recommendation for whole grains intake is ≥6 servings/day for a 2000 kcal diet. Thus, individuals who consumed ≥6 servings/day received a score of 1. Individuals who consumed ≥3 and <6 servings/day received a score of 0.5. Individuals who consumed less than 3 servings/day of whole grains received a score of 0. To assess adequacy of vitamin B-12 from reliable sources, we first estimated the average B-12 intake from foods known to contain vitamin B-12 (i.e., meats, fish, dairy (i.e., milk and cheese), eggs, yeast, meat substitutes, soymilk, supplements, and fortification from cereals). We then converted average intake to serving-equivalents of vitamin B-12, and defined adequate intake from reliable sources if intake met the Estimated Average Requirement (EAR) of 2.0 mcg per day. Thus, individuals who consumed ≥2.0 mcg serving equivalents of vitamin B-12 per day received 1 point. Individuals who consumed ≥1.0 and <2.0 mcg serving equivalents of vitamin B-12 per day received 0.5 points. Individuals who consumed <1.0 mcg serving equivalents of vitamin B-12 per day received 0 points. 

Reverse scoring was applied for components whose recommendations were to be consumed sparingly or in moderate amounts, such that higher intakes of these foods received lower scores. For example, individuals who consumed >5 servings/week of sweets received 0 points. Individuals who consumed >2 and ≤5 servings/week received 0.5 points. Individuals who consumed 0–2 servings/week received 1 point. For sweets, where there were no clear recommendations, cut-off scores were based on the probability density distribution to determine a relatively even distribution over a minimum and maximum range of scores. Similarly, intake of the flesh-food component was based on the frequency or avoidance of meat products. Individuals who consumed >1 time per week of flesh-food (including fish) received 0 points; those who consumed ≤1 time per week and >1 time per month received 0.5 points, and those who consumed ≤1 time per month of flesh-food (including fish) received 1 point. Table 1 shows the operationalization of the dietary and lifestyle components and their corresponding descriptions. 

Lastly, a composite score for the population was calculated as the sum of the scores obtained from the 14 components, ranging from 0–14 points. All components were equally weighted. Higher total scores reflect greater adherence to the LLU Vegetarian Food Guide Pyramid recommendations.

### 2.5. Statistical Analysis

VLI score was compared according to selected demographic variables. Analysis of variance (ANOVA) was conducted to obtain descriptive statistics and unadjusted mean scores according to demographic and lifestyle characteristics of the population. Interquartile range (IQR) and global *p*-trend tests were conducted where appropriate for selected demographic variables. We used a modeling approach to determine mean scores across dietary patterns, with dietary pattern as the independent variable and index scores as the dependent variable in Model 1. Analysis of covariance (ANCOVA) was performed to compare the adjusted means of index scores by categories of dietary pattern at 95% confidence interval (95% CI). Model 2 was adjusted for gender, age, race/ethnicity, and family history of cancer. Model 3 was adjusted as in Model 2 plus body mass index (BMI), smoking status, current alcohol use. Model 4 was adjusted as in Model 3 plus marital status, household income, and educational level. Additionally, an individual component analysis was conducted independently for the 14 diet and lifestyle components with non-vegetarians as the reference group. Assessment of normality, outliers, multi-collinearity, and Levene’s test for homogeneity of variance (HOV) were conducted. When homoscedasticity assumption was violated, general linear model (PROC GLM) for least squares procedures was performed. A *p*-value of <0.05 was considered statistically significant for all statistical tests. All analyses were conducted using SAS 9.4 software (SAS Institute, Inc., Cary, North CA, USA).

## 3. Results

Table 1 shows the index scoring criteria and frequency distributions by dietary and lifestyle components among an analytic sample of 90,057 participants. For adequacy components, the majority of participants received a score of 0.5 or 1.0, except for whole grains and vegetables. Nearly 77% of the cohort consumed <3 servings/day of whole grains, and more than half consumed <4 servings/day of vegetables. For components with reversed scoring such as vegetable oils and eggs, a high percentage of participants scored 0.5 or 1.0. In contrast, 74.5% of participants received a score of 0 or 0.5 for dairy products, and most received 0 points for flesh-food intake. For lifestyle components, majority of participants received a score of 0.5 or greater.

Table 2 shows the adherence scores according to selected characteristics among Adventists. The mean score (SD) for our population was 7.43 (1.75) ranging from 1.0 to 12.5 with interquartile range around 2.50 and 3.00. Participants with higher scores tended to be females, older than those aged 50 years (compared to <50 years), white (compared to other ethnicities), have a family history of cancer (compared to those with no family history of cancer), have normal BMI (compared to overweight and obese individuals), and were never or were past users of cigarette or alcohol (compared to users). Those with higher scores also tended to be currently married, have education level higher than high school, and have higher household income. Additionally, a global *p*-trend test showed a step-wise increase in score with increasing age, BMI, smoking and alcohol use, household income, and educational level.

**Figure 2:**
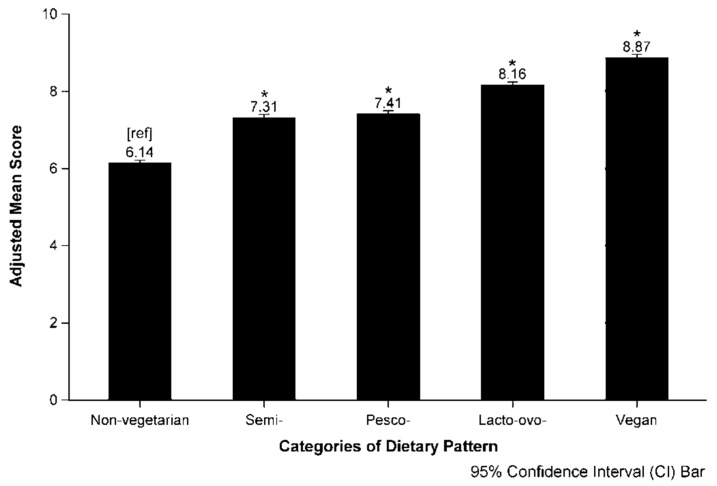
Vegetarian Lifestyle Index score according to categories of dietary pattern in the Adventist Health Study-2 (AHS-2) population. Adjusted for gender, race/ethnicity, family history of cancer, BMI, smoking status, alcohol use, marital status, household income, and educational level. * Significant difference between categories of dietary pattern with non-vegetarian as the reference *[ref]* group.

Table 3 reports the adjusted and unadjusted mean scores by categories of dietary pattern. Among the 90,057 participants, 47.7% were non-vegetarians, 5.6% were semi-vegetarians, 10.1% were pesco-vegetarians, 29.0% were lacto-ovo-vegetarians, and 7.7% were vegans. Non-vegetarians (6.38; 95% CI, 6.36–6.39) had a significantly lower mean score than the vegetarian groups, including semi- (7.61; 95% CI, 7.58–7.65), pesco- (7.78; 95% CI, 7.75–7.81), and lacto-ovo-vegetarians (8.54; 95% CI, 8.52–8.55), as well as vegans (9.27; 95% CI, 9.24, 9.30). Some attenuation occurred after adjusting for non-modifiable, lifestyle, and social-economic factors in Model 4: non-vegetarians (6.19; 95% CI, 6.12–6.25) had significantly lower mean compared to semi- (7.34; 95% CI, 7.27–7.42), pesco- (7.44; 95% CI, 7.37–7.51), lacto-ovo-vegetarians (8.20; 95% CI, 8.13–8.26), and vegans (8.88; 95% CI, 8.81–8.96). Figure 2 shows the dose-dependent relationship of mean scores across categories of dietary pattern. Table A1 (in Appendix A) reports the difference between vegetarians and non-vegetarians according to individual components. Vegetarians tended to score higher than their non-vegetarian counterparts on all the components, except with respect to dairy products and adequate sunlight exposure. 

## 4. Discussion

The VLI has strong discriminant ability across distinct categories of dietary pattern. Vegetarians scored, on average 1.18 to 2.73 points higher than non-vegetarians. Specifically, vegans had the highest, and non-vegetarians had the lowest scores, with a strong dose–response relationship across levels of dietary patterns from non-vegetarian, semi-, pesco-, and lacto-ovo-vegetarian, and vegan. The difference in observed scores between vegetarian patterns reflects dietary and lifestyle practices independent of age, gender, race and ethnicity, and socio-economic status. Vegetarians, specifically vegans, had the highest adherence to the LLU Vegetarian Food Guide Pyramid recommendations.

Of the 14 VLI components, 5 items were related to plant-based foods; one on nutrients (i.e., vitamin B-12); four were optional and to be consumed in moderate amounts; and three were related to lifestyle practices. Additionally, one of the 14 components related to meat intake was implicitly included to assess non-vegetarians in the overall population. At least 1 point difference can be explained by flesh-food intake component in favor of vegetarians. More specifically, vegans had 2.73 points more than non-vegetarians. This may be due to differences other than the absence of meat consumption. An individual component analysis (Appendix A, Table A1) showed an expected 0.95-point contribution to the difference between the vegan and non-vegetarian group. The remaining 1.78 points were due to combinations of dietary components, with lifestyle items modestly contributing to remaining difference. The VLI included some measures which are related to the definition of dietary patterns such as the consumption of meat, including fish. Results from the individual component analysis seems to indicate that there would be significant differences between dietary groups even excluding these elements. This suggests a trend towards healthier choices among those on plant-based diets, as animal foods are restricted beyond what would be expected from simple caloric substitution. Overall, vegetarians tended to have better water intake and physical activity than non-vegetarians.

The VLI scores for our population were consistently near the means (7.43, SD = 1.75) with a narrow interquartile range of 2.5 to 3.0 across demographic variables such as gender, age, race, socioeconomic status (SES), and educational level. The homogenenity and trends in the expected direction suggest that there is dietary stability within a population who adopt an Adventist lifestyle. In contrast, the general population tends to exhibit large differences in diet. For example, younger adults tend to eat more sweets and fast food, and eat fewer whole grains, fruits, and vegetables [46]. Unhealthy eating and lifestyle patterns are the highest among the younger age group for both genders [47]. Males are more likely to report eating meat and poultry, whereas females are more likely to report eating fruits and vegetables according to the FoodNet Population Survey [48,49]. Groups with higher socio-economic status have better nutrition knowledge and beliefs, greater awareness of nutrition-related health risks, and thus make better food choices [50]. 

Variability of the VLI scores between vegetarian and non-vegetarian Adventists may be due to notable differences in nutritional intake. Vegetarian diets tend to emphasize the consumption of plant-based foods (whole grains; legumes, soy, and meat substitutes; fruits and vegetables; nuts and seeds) which is reflected in the LLU Vegetarian Food Guide Pyramid. Similarly, the European Prospective Investigation into Cancer and Nutrition (EPIC)-Oxford cohort [51] showed comparable differences in nutrient profiles between vegetarians and non-vegetarians. In general, vegetarian diets are higher in carbohydrates from fruits, vegetables, whole grains, and dietary fibers; but lower in proteins and saturated fat [52,53]. Food displacement of animal products by these foods, which are characteristic of plant-based diets, may explain why vegetarians tend to have a better nutrient profile [54]. 

A recent study by Clarys et al. reported higher scores, on average, for vegetarians compared to non-vegetarians using the Healthy Eating Index (HEI) 2010 and Mediterranean Diet Score (MDS) as dietary indicators. Individual component analysis indicates that vegans obtain higher scores for vegetables and legumes [20,55]. Other less healthy sources of sugar such as sweets, candy, chocolate, cake, and beverages are consumed in limited amounts in vegans [56,57]. In most studies, macro- and micro-nutrient profile for the intake of vegetarians is much closer to the recommendations [58,59]. 

Compared to the HEI and MDS [20], the VLI has a relatively greater magnitude of difference in the effect size when comparing vegetarian to non-vegetarian groups. The WCRF/AICR index is the only other known index to incorporate both diet and lifestyle recommendations. However, epidemiologic studies using the WCRF scores have been limited to evaluating the incidence of cancer [60,61,62] and cancer-specific mortality [63,64]. Distinct from WCRF index, the VLI incorporates vegetarian diet and lifestyle behaviors which appear to discriminate between dietary patterns. Thus, the VLI can be applied in future studies to evaluate lifestyle–disease relationships.

The development of the index involved a number of key design decisions related to the choice of components, cut-off values, and their scoring criteria based on available scientific literature. For example, the choice to include flesh-food intake as one of the 14 components, although not in the VFGP recommendations, was implicitly added to assess non-vegetarians in the full cohort. Additionally, the VLI is largely based on food and food groups, while most nutrients were not analyzed directly. This is done for interpretation and ease of recommendations in the context of public health policy. Components whose dietary recommendations were to be consumed sparingly or in moderate amounts were operationalized using reversed scoring. For these components, it is less clear where to assign a zero score than it is for the adequacy components since higher levels of intake are given lower scores. The reverse scoring has no obvious mathematical basis that can be equivalent to zero in the adequacy components, and no scientific evidence specifying how high an intake deserves a score of zero. If the cutoff points were to be set disproportionally such that a majority of the population would get a zero, the index would not be sensitive to detect differences among individuals and groups. To mitigate this problem, a population density distribution was examined to determine the standards for minimum and maximum cut-off scores. 

Further considerations were made during the design and development of VLI to include a range of scores from low (0), moderate (0.5), and high (1.0)—instead of simple low-high cut-off values. This approach allows the total score to better represent a degree of compliance for individuals with intakes near the cut-off points. Additionally, recommended average daily intake amounts of selected foods and nutrients were standardized per 2000 kcal/day to avoid confounding effects by total energy [65]. Indices that include additional information on total food consumed in proportion to energy intake enhance the performance of the overall index [66]. 

### Strengths and Limitations

A notable strength of this study is the large proportion of participants who adhered to the vegetarian lifestyle. The shared religious affiliations of this cohort may lead to greater homogeneity (as seen in the stability of the mean index scores across demographic variables) and possible reduction of unmeasured confounders; thus, enhancing internal validity. Additionally, the precise definitions of ‘vegetarian’ and ‘non-vegetarian’ are based on measured food intake rather than self-identification. This may offer some advantage for consistency in evaluating the VLI scores to discriminate between dietary patterns. The FFQ was extensively tested to evaluate its ability to measure usual dietary intake, thus lending to its greater precision and accuracy.

One important limitation where caution is warranted is the generalizability of the Adventist lifestyle to other populations. The VLI did not include an additional component on cigarette smoking and alcohol use. Thus, this index may not be translational to other populations where there is a high prevalence of smokers and alcohol users. Additionally, the results from this study population may not be fully generalizable to other ethnic and cultural groups with different motivational attitudes, whose diet and lifestyle practices are markedly differently from the American norms. Diet patterns and lifestyle behaviors may change over time, whereas epidemiological measurement tools may only be able to capture a segment of the data. For VLI, all components are equally weighted. However, equally weighted ordinal scores tend to be biased toward a discriminant power loss. If the component were to be unequally weighted, there would be an expected greater difference in VLI scores across dietary patterns. Furthermore, other possible confounders may have been overlooked since people on vegetarian diets are often more health-conscious compared to the general population. 

## 5. Conclusions

Overall, our results indicate that the Vegetarian Lifestyle Index is a robust indicator of compliance to a healthy vegetarian lifestyle among a low-risk population (i.e., low prevalence of smoking and alcohol users). The VLI provides a useful epidemiologic measure of vegetarian diet and lifestyle adherence to refine future guidelines for more accurate recommendations given the shift towards non-smoking and reduced alcohol consumption in the general population. Looking at a global index in the context of LLU Vegetarian Food Guide Pyramid guidelines could enhance our conceptual understanding of dietary choices and lifestyle practices. A number of useful applications could be applied in nutritional research, including: evaluation of diet and lifestyle adherence in community based-interventions, population-level surveillance of diet-lifestyle quality over time, and epidemiological investigations of disease outcomes in other populations such as the EPIC-Oxford cohort. We anticipate that the VLI could be applied to future prospective studies to assess incidence of diabetes, cardiovascular disease, and mortality outcomes. Furthermore, the global diet–lifestyle index has several important public health implications, including the ease and simplicity of interpretation to be translated into plausible diet and lifestyle recommendations. Clinicians and health professions could potentially develop nutritional interventions and educational guidance to target improvements in the most critical aspects of the individual’s or population’s dietary intake and lifestyle choices. By exploring diet–lifestyle adherence in a population, key areas of concern could be identified to provide evidence-based guidance for development of policies and prevention efforts in the promotion of population health.

## Figures and Tables

**Table 1:** Operationalization of Vegetarian Lifestyle Index (VLI) based on the LLU-Vegetarian Food Guide Pyramid Recommendations.

Component	Diet/Lifestyle Description	Recommendation	Operationalization	Score	Frequency	%
**1: Whole grains**	Whole grain bread, cereals such as oatmeal, and brown rice	6 servings/day per 2000 kcal	<3 servings/day	**0**	69,456	77.1
			≥3 and <6 servings/day	**0.5**	19,876	22.1
			≥6 servings/day	**1**	725	0.8
**2:** **Legumes, soy, and meat substitutes**	Beans, peas, soy, and meat substitutes	3 servings/day per 2000 kcal	<1 servings/day	**0**	25,339	28.1
			≥1 and <3 servings/day	**0.5**	47,490	52.7
			≥3 serving/day	**1**	17,228	19.1
**3: Vegetables**	Dark green vegetables, avocado, and 100% vegetable juice	8 servings/day per 2000 kcal	<4 servings/day	**0**	45,524	50.6
			≥4 and <8 servings/day	**0.5**	38,310	42.5
			≥8 serving/day	**1**	6223	6.9
**4: Fruits**	Fresh and dried fruits, canned or cooked fruits, and 100% fruit juices	4 servings/day per 2000 kcal	<2 servings/day	**0**	21,443	23.8
			≥2 and <4 servings/day	**0.5**	35,586	39.5
			≥4 serving/day	**1**	33,028	36.7
**5: Nuts and seeds**	Nuts and seeds (raw or roasted)	1–2 serving/day per 2000 kcal	<4 servings/week	**0**	25,417	28.2
			≥4 servings/week and <1.5 servings/day	**0.5**	33,261	36.9
			≥1.5 serving/day	**1**	31,379	34.8
**6: Vegetable oils ^‡^**	Olive oil, and other salad oils	2 serving/day per 2000 kcal	>4 servings/day	**0**	4755	5.3
			>2 and ≤4 servings/day	**0.5**	28,455	31.6
			0–2 servings/day	**1**	56,847	63.1
**7: Dairy products ^‡^**	Dairy products, cheese, milk, and yogurt	2 servings/day per 2000 kcal	<0.5 or >2 servings/day	**0**	52,484	58.3
			≥0.5 and <1 servings/day	**0.5**	14,561	16.2
			1–2 servings/day	**1**	23,012	25.6
**8: Eggs ^‡^**	Eggs (fried, boiled, scrambled deviled, plain omelet, egg salad)	1/2 servings/day per 2000 kcal	>1 servings/day	**0**	3263	3.6
			>0.5 and ≤1 servings/day	**0.5**	11,483	12.8
			≥0 and ≤0.5 servings/day	**1**	75,311	83.6
**9: Sweets ^‡ $^**	Sweets, sugary beverages, and desserts	Consume sweets and sugary beverages sparingly, per 2000 kcal	>5 servings/week	**0**	35,877	39.8
			>2 and ≤5 servings/week	**0.5**	23,809	26.4
			0–2 servings/week	**1**	30,371	33.7
**10: Reliable sources of vitamin B-12**	Reliable sources of vitamin B-12 from reliable sources including meat, fish, dairy, eggs; yeast, fortification (cereals, meat substitutes, soymilk); supplementations	Meeting the recommended Estimated Average Requirement (EAR) of 2.0 mcg daily of vitamin B-12, based on per 2000 kcal	<1.0 mcg serving equivalent/day	**0**	11,008	12.2
			≥1.0 and <2.0 mcg serving equivalent/day	**0.5**	24,599	27.3
			≥2.0 mcg serving equivalent/day	**1**	54,450	60.5
**11: Flesh-food intake ^‡^**	Red meat, processed meat, poultry, and fish		>1 time/week	**0**	44,898	49.9
			≤1 time/week and >1 time/month	**0.5**	11,521	12.8
			≤1 time/month	**1**	33,638	37.4
**Lifestyle Component**						
**12:** **Daily exercise ^¥^**	Moderate/rigorous physical activity	30 min/day to avoid chronic disease, and 60 min/day for weight loss	≤0 min/day of moderate OR ≤0 min/day of vigorous exercise	**0**	18,949	21.0
			>0 and <30 min/day of moderate exercise OR >0 and <15 min/day of vigorous exercise	**0.5**	66,608	74.0
			≥30 min/day of moderate OR ≥15 min/day of vigorous exercise	**1**	4500	5.0
**13:** **Water intake**	Drinking water	At least eight, 8-oz glasses of water daily, per 2000 kcal	<4 glasses of water/day	**0**	33,616	37.3
			≥4 and <8 glasses of water/day	**0.5**	37,276	41.4
			≥8 glasses of water/day	**1**	19,165	21.3
**14:** Sunlight exposure *	Adequate exposure to sunlight	At least 10 min of sun a day to activate vitamin D	<5 min/day	**0**	2436	2.7
			≥5 and <10 min/day	**0.5**	725	0.8
			≥10 min/day	**1**	86,896	96.5

^‡^ Reverse scoring was operationalized for components whose dietary recommendations were to be consumed sparingly or in moderate amounts. Higher intakes of these foods received lower scores. All others are adequacy components: whole grains; legumes, soy, and meat substitutes; vegetables; fruits; nuts and seeds; reliable sources of vitamin B-12; daily exercise; water intake; sunlight exposure. ^$^ Optional: no specific recommendations from the Loma Linda University Vegetarian Food Guide Pyramid. ^¥^ Daily exercise is defined as moderate activity (such as walking, running, or jogging); vigorous activity (with enough intensity to work up a sweat, get the heart thumping or get out of breath). * Time exposure to direct sunlight during warmer months (April–September) and cooler months (October–March) between 9 a.m. and 5 p.m.

**Table 2:** Mean VLI score according to selected demographic characteristics in the Adventist Health Study-2 (AHS-2) population.

	No. of Participants (%)	Mean	*±*SD	P_25_	P_75_	IQR	*p*-Value	*p*-Trend
**Participant characteristics, scores**	90,057 (100%)	7.43	1.75	6.00	9.00	3.00	-	-
**Gender**								<0.0001
Female	58,265 (64.7)	7.48	1.75	6.00	9.00	3.00	*[ref]*	
Male	31,792 (35.3)	7.36	1.75	6.00	8.50	2.50	<0.0001	
**Age**								<0.0001
Less than 50 years	28,615 (31.8)	7.22	1.76	6.00	8.50	2.50	*[ref]*	
50–64 years	29,372 (32.6)	7.46	1.76	6.00	9.00	3.00	<0.0001	
Greater than 64 years	32,070 (35.6)	7.61	1.72	6.50	9.00	2.50	<0.0001	
**Race**								
Non-black	66,557 (74.6)	7.55	1.77	6.50	9.00	2.50	*[ref]*	
Black	22,703 (25.4)	7.09	1.66	6.00	8.50	2.50	<0.0001	
**Ethnicity**								
White	59,259 (66.4)	7.57	1.78	6.50	9.00	2.50	*[ref]*	
Black	22,481 (25.2)	7.09	1.66	6.00	8.50	2.50	<0.0001	
Hispanic	3485 (3.9)	7.32	1.73	6.00	8.50	2.50	<0.0001	
Middle Eastern	120 (0.1)	7.59	1.65	6.50	9.00	2.50	0.9158	
Asian	2705 (3.0)	7.53	1.58	6.50	8.50	2.00	0.2749	
Hawaiian	94 (0.1)	6.86	1.71	5.50	8.00	2.50	<0.0001	
American Indian	323 (0.4)	7.02	1.81	5.50	8.50	3.00	<0.0001	
Mixed	793 (0.9)	7.31	1.74	6.00	8.50	2.50	<0.0001	
**Family history of cancer**								<0.0001
No	5222 (5.8)	7.33	1.73	6.00	8.50	2.50	<0.0001	
Yes	84,835 (94.2)	7.44	1.76	6.00	9.00	3.00	*[ref]*	
**BMI ^‡^**								<0.0001
Underweight	1682 (1.9)	8.06	1.72	7.00	9.50	2.50	<0.0001	
Normal weight	35,225 (39.1)	7.87	1.69	6.50	9.00	2.50	*[ref]*	
Overweight	31,322 (34.8)	7.33	1.71	6.00	8.50	2.50	<0.0001	
Obese	21,828 (24.2)	6.83	1.72	5.50	8.00	2.50	<0.0001	
**Smoking status**								<0.0001
Never	72,129 (80.1)	7.55	1.72	6.50	9.00	2.50	*[ref]*	
In the past	16,945 (18.8)	7.03	1.79	5.50	8.50	3.00	<0.0001	
Current	983 (1.1)	5.54	1.54	4.50	6.50	2.00	<0.0001	
**Alcohol use**								<0.0001
Never	53,482 (59.4)	7.70	1.69	6.50	9.00	2.50	*[ref]*	
In the past	27,706 (30.8)	7.23	1.78	6.00	8.50	2.50	<0.0001	
Current	8869 (9.9)	6.44	1.62	5.50	7.50	2.00	<0.0001	
**Marital status**								
Never married	5641 (6.3)	7.17	1.77	6.00	8.50	2.50	<0.0001	
Currently married	65,021 (72.2)	7.49	1.75	6.00	9.00	3.00	*[ref]*	
Married in the past	19,395 (21.5)	7.31	1.75	6.00	8.50	2.50	<0.0001	
**Household income, $USD per year**								<0.0001
Less than 10,000	5641 (6.3)	7.17	1.77	6.00	8.50	3.00	*[ref]*	
10,000 to 75,000	65,021 (72.2)	7.49	1.75	6.00	9.00	2.50	0.2738	
>75,000 to 200,000	19,395 (21.5)	7.31	1.75	6.00	9.00	2.50	0.1444	
More than 200,000	5641 (6.3)	7.17	1.77	6.50	9.00	2.00	0.5958	
**Education level**								<0.0001
High school or less	19,508 (21.7)	7.10	1.78	6.00	8.50	2.50	*[ref]*	
Trade school, associate degree, or some college	35,560 (39.5)	7.37	1.77	6.00	8.50		<0.0001	
Bachelor degree	18,976 (21.1)	7.62	1.70	6.50	9.00	2.50	<0.0001	
Graduate degree	16,013 (17.8)	7.78	1.66	6.50	9.00	2.50	<0.0001	

Abbreviations: BMI, body mass index (calculated as weight in kilograms divided by height in meters squared). Marital status is defined as never married, currently married (first marriage, remarried, common law married), or married in the past (separated, divorced, widowed). ^‡^ BMI categories are defined as underweight (<18.8), normal (18.5–24.9), overweight (25–29.9), and obese (≥30). IQR, inter-quartile range, is defined as 25th percentile (P_25_)–75th percentile (P_75_). A global *p*-trend test with *p* < 0.05 indicates statistical significance, and *[ref]* is the reference group.

**Table 3:** Mean Vegetarian Lifestyle Index (VLI) score according to dietary patterns in the Adventist Health Study-2 (AHS-2) cohort.

		*Unadjusted Model*		*Adjusted Model*							
		1		2		3			4		
**Dietary pattern**	*n* (%)	Mean (±SD)	95% CI	Mean	95% CI		Mean	95% CI		Mean	95% CI
**Non-vegetarian**	42,954 (47.7)	6.38 (1.48)	6.36, 6.39	6.45	6.40, 6.51		6.15	6.09, 6.21		6.14	6.06, 6.21
**Vegetarian**											
***Semi-***	5,007 (5.6)	7.61 (1.42)	7.58, 7.65	7.69	7.62, 7.76		7.31	7.24, 7.38		7.31	7.22, 7.40
***Pesco-***	9,078 (10.1)	7.78 (1.34)	7.75, 7.81	7.83	7.77, 7.89		7.42	7.35, 7.49		7.41	7.32, 7.49
***Lacto-ovo-***	26,101 (29.0)	8.54 (1.30)	8.52, 8.55	8.63	8.57, 8.68		8.18	8.12, 8.25		8.16	8.08, 8.24
***Vegan***	6,917 (7.7)	9.27 (1.05)	9.24, 9.30	9.35	9.29, 9.42		8.85	8.78, 8.92		8.87	8.78, 8.96

Model 1: Unadjusted model. Model 2: Adjusted for gender, age, race/ethnicity, and family history of cancer. Model 3: Adjusted as in Model 2 + BMI, smoking status, and alcohol use. Model 4: Adjusted as in Model 3 + marital status, household income, and educational level. Marginal (adjusted) means were reported for analysis of covariance (ANCOVA) models 2, 3, and 4. A global *p*-trend test with *p* < 0.05 indicates statistical significance. Model 1, 2, 3, and 4 (*p*-trend < 0.0001)

## Appendix A

**Table A1:** Mean difference of VLI scores between non-vegetarian and vegetarian groups according to individual components.

	Categories of Dietary Pattern													
Components	Non-Vegetarian			Semi-		Pesco-			Lacto-Ovo-			Vegan		
	Mean	*[ref]*	Mean	*[Diff]*	*SE*	Mean	*[Diff]*	*SE*	Mean	*[Diff]*	*SE*	Mean	*[Diff]*	*SE*
**1: Whole grains**	0.05	*[ref]*	0.10	*0.04*	0.004	0.08	0.03	0.003	0.11	*0.06*	0.002	0.20	*0.15*	0.003
**2: Legumes, soy, meat substitutes**	0.29	*[ref]*	0.47	*0.18*	0.006	0.53	0.24	0.004	0.55	*0.26*	0.003	0.58	*0.29*	0.005
**3: Vegetables**	0.24	*[ref]*	0.27	*0.03*	0.005	0.32	0.08	0.004	0.28	*0.04*	0.003	0.40	*0.16*	0.005
**4: Fruits**	0.44	*[ref]*	0.51	*0.06*	0.007	0.56	0.12	0.005	0.54	*0.10*	0.004	0.69	*0.25*	0.006
**5: Seeds and nuts**	0.42	*[ref]*	0.48	*0.05*	0.007	0.53	0.11	0.005	0.54	*0.12*	0.004	0.68	*0.26*	0.006
**6: Vegetable oils**	0.76	*[ref]*	0.77	*0.01*	0.005	0.77	0.00	0.004	0.79	*0.02*	0.003	0.83	*0.07*	0.005
**7: Dairy products**	0.41	*[ref]*	0.40	*−0.02*	0.008	0.35	−0.07	0.006	0.34	*−0.08*	0.004	0.02	*−0.39*	0.007
**8: Eggs**	0.86	*[ref]*	0.91	*0.05*	0.004	0.92	0.06	0.003	0.93	*0.07*	0.002	0.99	*0.13*	0.004
**9: Sweets**	0.34	*[ref]*	0.44	*0.11*	0.007	0.55	0.21	0.006	0.51	*0.17*	0.004	0.78	*0.44*	0.006
**10: Reliable sources of vitamin B-12**	0.63	*[ref]*	0.74	*0.11*	0.006	0.79	0.16	0.005	0.80	*0.18*	0.003	0.82	*0.19*	0.005
**11: Flesh food intake**	0.02	*[ref]*	0.47	*0.44*	0.002	0.27	0.25	0.002	0.98	*0.95*	0.001	0.98	*0.95*	0.002
**12: Exercise**	0.40	*[ref]*	0.40	*0.01*	0.004	0.42	0.02	0.003	0.41	*0.01*	0.002	0.42	*0.02*	0.004
**13: Water intake**	0.30	*[ref]*	0.40	*0.10*	0.007	0.37	0.06	0.005	0.41	*0.11*	0.004	0.52	*0.22*	0.006
**14: Sunlight exposure**	0.96	*[ref]*	0.95	*−0.01*	0.003	0.96	0.00	0.002	0.95	*−0.01*	0.002	0.95	*−0.01*	0.003
**Total score**	**6.14**	*[ref]*	**7.31**	**1.18**	0.075	**7.41**	**1.27**	0.06	**8.16**	**2.03**	0.041	**8.87**	**2.73**	0.066

[Diff] = Difference of VLI scores between non-vegetarian (as the reference, *[ref]*) and vegetarian groups. SE = standard error of the mean difference.

## References

[1] Madden J.P., Yoder M. (1972). Program Evaluation: Food Stamps and Commodity Distribution in Rural Areas of Central Pennsylvania.

[2] Hansen R.G. (1973). An index of food quality. Nutr. Rev..

[3] Clarke M., Wakefield L.M. (1975). Food choices of institutionalized vs. independent-living elderly. J. Am. Diet. Assoc..

[4] Hulshof K.F., Wedel M., Löwik M.R., Kok F.J., Kistemaker C., Hermus R.J., Ockhuizen T. (1992). Clustering of dietary variables and other lifestyle factors (Dutch nutritional surveillance system). J. Epidemiol. Commun. Health.

[5] Davis M.A., Murphy S.P., Neuhaus J.M., Gee L., Quiroga S.S. (2000). Living arrangements affect dietary quality for U.S. adults aged 50 years and older: NHANES III 1988–1994. J. Nutr..

[6] Toft U., Kristoffersen L.H., Lau C., Borch-Johnsen K., Jørgensen T. (2007). The dietary quality score: Validation and association with cardiovascular risk factors: The inter99 study. Eur. J. Clin. Nutr..

[7] Davenport M., Roderick P., Elliott L., Victor C., Geissler C. (1995). Monitoring dietary change in populations and the need for specific food targets; lessons from the North West Thames regional health survey. J. Hum. Nutr. Diet..

[8] Randall E., Marshall J.R., Graham S., Brasure J. (1991). High-risk health behaviors associated with various dietary patterns. Nutr. Cancer.

[9] Ping-Delfos W.L.C.S., Beilin L.J., Oddy W.H., Burrows S., Mori T.A. (2015). Use of the Dietary Guideline Index to assess cardiometabolic risk in adolescents. Br. J. Nutr..

[10] Golley R.K., McNaughton S.A., Hendrie G.A. (2015). A dietary guideline adherence score is positively associated with dietary biomarkers but not lipid profile in healthy children. J. Nutr..

[11] Kant A.K., Schatzkin A., Graubard B.I., Schairer C. (2000). A prospective study of diet quality and mortality in women. JAMA.

[12] McCullough M.L., Feskanich D., Stampfer M.J., Giovannucci E.L., Rimm E.B., Hu F.B., Willett W.C. (2002). Diet quality and major chronic disease risk in men and women: Moving toward improved dietary guidance. Am. J. Clin. Nutr..

[13] Michels K.B., Wolk A. (2002). A prospective study of variety of healthy foods and mortality in women. Int. J. Epidemiol..

[14] Cespedes E.M., Hu F.B. (2015). Dietary patterns: From nutritional epidemiologic analysis to national guidelines. Am. J. Clin. Nutr..

[15] Gil A., Martinez de Victoria E., Olza J. (2015). Indicators for the evaluation of diet quality. Nutr. Hosp..

[16] Yu D., Sonderman J., Buchowski M.S., McLaughlin J.K., Shu X.O., Steinwandel M., Zheng W. (2015). Healthy eating and risks of total and cause-specific death among low-income populations of African-Americans and other adults in the Southeastern United States: A prospective cohort study. PLoS Med..

[17] Schwingshackl L., Hoffmann G. (2015). Diet quality as assessed by the healthy eating index, the alternate healthy eating index, the dietary approaches to stop hypertension score, and health outcomes: A Systematic review and meta-analysis of cohort studies. J. Acad. Nutr. Diet..

[18] Xie J., Poole E.M., Terry K.L., Fung T.T., Rosner B.A., Willett W.C., Tworoger S.S. (2014). A prospective cohort study of dietary indices and incidence of epithelial ovarian cancer. J. Ovarian Res..

[19] Jacobs S., Harmon B.E., Boushey C. J., Morimoto Y., Wilkens L.R., Le Marchand L., Maskarinec G. (2015). A priori-defined diet quality indexes and risk of type 2 diabetes: The multiethnic cohort. Diabetologia.

[20] Clarys P., Deliens T., Huybrechts I., Deriemaeker P., Vanaelst B., De Keyzer W., Mullie P. (2014). Comparison of nutritional quality of the vegan, vegetarian, semi-vegetarian, pesco-vegetarian and omnivorous diet. Nutrients.

[21] Harmon B.E., Boushey C.J., Shvetsov Y.B., Ettienne R., Reedy J., Wilkens L.R., Kolonel L.N. (2015). Associations of key diet-quality indexes with mortality in the multiethnic cohort: The dietary patterns methods project. Am. J. Clin. Nutr..

[22] Liese A.D., Krebs-Smith S.M., Subar A.F., George S.M., Harmon B.E., Neuhouser M.L., Reedy J. (2015). The dietary patterns methods project: Synthesis of findings across cohorts and relevance to dietary guidance. J. Nutr..

[23] Malagoli C., Malavolti M., Agnoli C., Crespi C.M., Fiorentini C., Farnetani F., Veneziano L. (2015). Diet quality and risk of melanoma in an Italian population. J. Nutr..

[24] Sotos-Prieto M., Bhupathiraju S.N., Mattei J., Fung T.T., Li Y., Pan A., Hu F.B. (2017). Association of changes in diet quality with total and cause-specific mortality. N. Engl. J. Med..

[25] Haveman-Nies A., de Groot L.C., van Staveren W.A. (2003). Dietary quality, lifestyle factors and healthy ageing in Europe: The SENECA study. Age Ageing.

[26] Zeng F.F., Xue W.Q., Cao W.T., Wu B.H., Xie H.L., Fan F., Chen Y.M. (2014). Diet-quality scores and risk of hip fractures in elderly urban Chinese in Guangdong, China: A case-control study. Osteoporos. Int..

[27] Mila-Villarroel R., Bach-Faig A., Puig J., Puchal A., Farran A., Serra-Majem L., Carrasco J.L. (2011). Comparison and evaluation of the reliability of indexes of adherence to the Mediterranean diet. Public Health Nutr..

[28] Makarem N., Lin Y., Bandera E.V., Jacques P.F., Parekh N. (2015). Concordance with world cancer research fund/American institute for cancer research (WCRF/AICR) guidelines for cancer prevention and obesity-related cancer risk in the Framingham offspring cohort (1991–2008). Cancer Causes Control.

[29] Romaguera D., Vergnaud A.C., Peeters P.H., van Gils C.H., Chan D.S., Ferrari P., Fagherazzi G. (2012). Is concordance with world cancer research fund/American institute for cancer research guidelines for cancer prevention related to subsequent risk of cancer? Results from the EPIC study. Am. J. Clin. Nutr..

[30] Cerhan J.R., Potter J.D., Gilmore J.M., Janney C.A., Kushi L.H., Lazovich D., Folsom A.R. (2004). Adherence to the AICR cancer prevention recommendations and subsequent morbidity and mortality in the Iowa women’s health study cohort. Cancer Epidemiol. Biomarkers Prev..

[31] Inoue-Choi M., Lazovich D., Prizment A.E., Robien K. (2013). Adherence to the world cancer research fund/American institute for cancer research recommendations for cancer prevention is associated with better health-related quality of life among elderly female cancer survivors. J. Clin. Oncol..

[32] Realdon S., Antonello A., Arcidiacono D., Dassie E., Cavallin F., Fassan M., Battaglia G. (2016). Adherence to WCRF/AICR lifestyle recommendations for cancer prevention and the risk of Barrett’s esophagus onset and evolution to esophageal adenocarcinoma: Results from a pilot study in a high-risk population. Eur. J. Nutr..

[33] Haddad E.H., Sabate J., Whitten C.G. (1999). Vegetarian food guide pyramid: A conceptual framework. Am. J. Clin. Nutr..

[34] Butler T.L., Fraser G.E., Beeson W.L., Knutsen S.F., Herring R.P., Chan J., Bennett H. (2008). Cohort profile: The adventist health study-2 (AHS-2). Int. J. Epidemiol..

[35] Jaceldo-Siegl K., Knutsen S.F., Sabaté J., Beeson W.L., Chan J., Herring R.P., Sharma S.S. (2010). Validation of nutrient intake using an FFQ and repeated 24 h recalls in black and white subjects of the adventist health study-2 (AHS-2). Public Health Nutr..

[36] Ness A. (2003). Diet Life Expectancy and Chronic Disease. Studies of Seventh-Day Adventists and Other Vegetarians. Gary E Fraser.

[37] Fraser G.E. (2009). Vegetarian diets: What do we know of their effects on common chronic diseases?. Am. J. Clin. Nutr..

[38] Barnard N.D., Katcher H.I., Jenkins D. J., Cohen J., Turner-McGrievy G. (2009). Vegetarian and vegan diets in type 2 diabetes management. Nutr. Rev..

[39] Barnard N.D., Levin S.M., Yokoyama Y. (2015). A systematic review and meta-analysis of changes in body weight in clinical trials of vegetarian diets. J. Acad. Nutr. Diet..

[40] Yokoyama Y., Barnard N.D., Levin S.M., Watanabe M. (2014). Vegetarian diets and glycemic control in diabetes: A systematic review and meta-analysis. Cardiovasc. Diagn. Ther..

[41] Yokoyama Y., Nishimura K., Barnard N.D., Takegami M., Watanabe M., Sekikawa A., Miyamoto Y. (2014). Vegetarian diets and blood pressure: A meta-analysis. JAMA Intern. Med..

[42] Ajala O., English P., Pinkney J. (2013). Systematic review and meta-analysis of different dietary approaches to the management of type 2 diabetes. Am. J. Clin. Nutr..

[43] Haddad E.H. (1994). Development of a vegetarian food guide. Am. J. Clin. Nutr..

[44] International Congress on Vegetarian Nutrition (ICVN) Loma Linda University Vegetarian Food Guide Pyramid 2012. http://www.vegetariannutrition.org/6icvn/food-pyramid.pdf.

[45] Willett W. (1998). Nutritional Epidemiology.

[46] Nicklett E.J., Kadell A.R. (2013). Fruit and vegetable intake among older adults: A scoping review. Maturitas.

[47] Kushner R.F., Choi S.W. (2010). Prevalence of unhealthy lifestyle patterns among overweight and obese adults. Obesity.

[48] Shiferaw B., Verrill L., Booth H., Zansky S.M., Norton D.M., Crim S., Henao O.L. (2012). Sex-Based differences in food consumption: Foodborne diseases active surveillance network (foodnet) population survey, 2006–2007. Clin. Infect. Dis..

[49] Baker A.H., Wardle J. (2003). Sex differences in fruit and vegetable intake in older adults. Appetite.

[50] Wang Y., Chen X. (2012). Between-group differences in nutrition-and health-related psychosocial factors among US adults and their associations with diet, exercise, and weight status. J. Acad. Nutr. Diet..

[51] Davey G.K., Spencer E.A., Appleby P.N., Allen N.E., Knox K.H., Key T.J. (2003). EPIC-Oxford: Lifestyle characteristics and nutrient intakes in a cohort of 33 883 meat-eaters and 31 546 non meat-eaters in the UK. Public Health Nutr..

[52] Rizzo N.S., Jaceldo-Siegl K., Sabate J., Fraser G.E. (2013). Nutrient profiles of vegetarian and nonvegetarian dietary patterns. J. Acad. Nutr. Diet..

[53] Craig W.J., Mangels A.R. (2009). Position of the American dietetic association: Vegetarian diets. J. Am. Diet. Assoc..

[54] Key T.J., Appleby P.N., Spencer E.A., Travis R.C., Roddam A.W., Allen N.E. (2009). Mortality in British vegetarians: Results from the European prospective investigation into cancer and nutrition (EPIC-Oxford). Am. J. Clin. Nutr..

[55] Clarys P., Deriemaeker P., Huybrechts I., Hebbelinck M., Mullie P. (2013). Dietary pattern analysis: A comparison between matched vegetarian and omnivorous subjects. Nutr. J..

[56] Larsson C.L., Johansson G.K. (2002). Dietary intake and nutritional status of young vegans and omnivores in Sweden. Am. J. Clin. Nutr..

[57] Orlich M.J., Jaceldo-Siegl K., Sabaté J., Fan J., Singh P.N., Fraser G.E. (2014). Patterns of food consumption among vegetarians and non-vegetarians. Br. J. Nutr..

[58] Deriemaeker P., Aerenhouts D., Hebbelinck M., Clarys P. (2010). Nutrient based estimation of acid-base balance in vegetarians and non-vegetarians. Plant Foods Hum. Nutr..

[59] Deriemaeker P., Aerenhouts D., De Ridder D., Hebbelinck M., Clarys P. (2011). Health aspects, nutrition and physical characteristics in matched samples of institutionalized vegetarian and non-vegetarian elderly (>65 yrs). Nutr. Metab..

[60] Turati F., Bravi F., Di Maso M., Bosetti C., Polesel J., Serraino D., Negri E. (2017). Adherence to the world cancer research fund/American institute for cancer research recommendations and colorectal cancer risk. Eur. J. Cancer..

[61] Romaguera D., Gracia-Lavedan E., Molinuevo A., de Batlle J., Mendez M., Moreno V., Molina A.J. (2017). Adherence to nutrition-based cancer prevention guidelines and breast, prostate and colorectal cancer risk in the MCC-Spain case-control study. Int. J. Cancer..

[62] Jankovic N., Geelen A., Winkels R.M., Mwungura B., Fedirko V., Jenab M., Franco O.H. (2017). Adherence to the WCRF/AICR dietary recommendations for cancer prevention and risk of cancer in elderly from Europe and the United States: A meta-analysis within the CHANCES project. Cancer Epidemiol. Biomarkers Prev..

[63] Hastert T.A., Beresford S.A., Sheppard L., White E. (2014). Adherence to the WCRF/AICR cancer prevention recommendations and cancer-specific mortality: Results from the vitamins and lifestyle (VITAL) study. Cancer Causes Control.

[64] Vergnaud A.C., Romaguera D., Peeters P.H., Van Gils C.H., Chan D.S., Romieu I., Dartois L. (2013). Adherence to the world cancer research fund/American institute for cancer research guidelines and risk of death in Europe: Results from the European prospective investigation into nutrition and cancer cohort study. Am. J. Clin. Nutr..

[65] Waijers P.M., Feskens E.J., Ocke M.C. (2007). A critical review of predefined diet quality scores. Br. J. Nutr..

[66] Fransen H.P., Ocke M.C. (2008). Indices of diet quality. Curr. Opin. Clin. Nutr. Metab. Care.

